# Ventral root evoked entrainment of disinhibited bursts across early postnatal development in mice

**DOI:** 10.1016/j.ibror.2020.10.005

**Published:** 2020-10-27

**Authors:** Chetan Nagaraja

**Affiliations:** Developmental Neurobiology Section, National Institute of Neurological Disorders and Stroke, National Institutes of Health, Bethesda, MD, 20892, United States

**Keywords:** D2, dopamine receptor subtype 2, DH, donepezil hydrochloride, DHPG, (RS)-3,5-dihydroxyphenylglycine, LLA, locomotor-like activity, mGluR1, metabotropic glutamate receptor subtype 1, P, postnatal day, TBOA, DL-Threo-β-Benzyloxyaspartic acid, Motoneurons, Spinal cord, Disinhibited bursting, Entrainment, Network function

## Abstract

Early in the postnatal period, motoneuron axon stimulation can excite motor networks in the spinal cord. Here we tested if these excitatory effects changed across early postnatal development up to postnatal day (P) 24 by when mice are capable of weight-bearing locomotion and locomotor networks are considered functionally mature. This was accomplished in the isolated spinal cord preparation using ventral root evoked entrainment of disinhibited bursts. Ventral root evoked entrainment was defined and characterized over the first 2 weeks of postnatal development, and was found to decline over this period, but entrainment could still be detected in mice as old as P24. Disinhibited bursting could be elicited, and dorsal root evoked entrainment could be recorded as late as P39 and remained unchanged in effectiveness, suggesting that poor tissue viability may not be the cause of the decline in ventral root evoked entrainment. Pharmacological experiments performed on younger animals established that dopamine D2 receptor antagonists and mGluR1 agonists both enhanced ventral root evoked entrainment. In conclusion, the motoneuronal inputs to spinal motor networks via the excitatory pathway is modulated by dopamine and metabotropic glutamate receptors and may be under powerful inhibitory control, which may explain why there is a developmental decline in entrainment.

## Highlights

• Ventral root evoked entrainment of disinhibited bursts can be elicited in P24 spinal cord preparations.
• Disinhibited bursting and dorsal root evoked entrainment can be elicited even at P39.
• Ventral root evoked entrainment shows a decline from P0−15, and the coefficient of variation increases during this period.
• Ventral root evoked entrainment decays after a trial and shows some recovery after long periods following a trial.
• Dopamine D2 receptor antagonists and mGluR1 agonists can enhance ventral root evoked entrainment.

## Introduction

In the spinal cord of the neonatal mouse, stimulation of motoneurons can excite the central pattern generator for locomotion (Mentis, Alvarez et al. 2005; Bonnot, Chub et al. 2009; Pujala, Blivis et al. 2016) and entrain disinhibited bursting (Mentis, Alvarez et al. 2005; Machacek and Hochman, 2006; Bonnot, Chub et al. 2009; Humphreys and Whelan, 2012). In addition, optogenetic experiments have revealed that manipulation of motoneuron discharge can regulate the frequency of locomotor-like activity generated by the isolated spinal cord (Falgairolle, Puhl et al. 2017). Motoneurons have recently been shown to make intraspinal connections with excitatory V3 glutamatergic interneurons (Chopek, Nascimento et al. 2018) in addition to inhibitory Renshaw cells (Renshaw, 1941, 1946, Eccles, Eccles et al., 1961; Wenner and O’Donovan, 1999; Alvarez and Fyffe, 2007; Bhumbra, Bannatyne et al. 2014). Several studies have shown that AMPA, NMDA and/or metabotropic glutamate receptors mediate many of the excitatory effects of motoneurons on neural elements of spinal motor networks (Mentis, Alvarez et al. 2005; Bonnot, Chub et al. 2009; Falgairolle, Puhl et al. 2017; Chopek, Nascimento et al. 2018). The excitatory effect of ventral root stimulations, in the presence of metabotropic glutamate receptor antagonists, has been shown to suppress entrainment of disinhibited bursting, and block locomotor-like activity from being evoked (Bonnot, Chub et al. 2009). In addition, Dopamine blocks the excitatory effects of ventral root stimulation on both disinhibited bursting and locomotor CPG, mediating this effect partly via the D2 receptors (Humphreys and Whelan, 2012).

Although it has been demonstrated that motoneuron postsynaptic AMPA and NMDA receptor actions on Renshaw cells and motoneurons are maintained in the adult (Lamotte d’Incamps, Bhumbra et al. 2017; Bhumbra and Beato, 2018), it is yet unclear if the excitatory effects of motoneurons on spinal motor networks are purely a developmental phenomenon; most studies on the isolated mouse spinal cord have been carried out during early neonatal period (P0-P4). To address this question, we have determined if the excitatory effects of ventral root stimulation can be detected up to P24. Using the isolated spinal cord preparation the oldest age at which complex motor activity has been recorded successfully is P15, when the mice is capable of weight-bearing locomotion (Jiang, Carlin et al. 1999). To examine the excitatory actions of motoneurons on spinal motor networks across early postnatal development we chose to study ventral root evoked entrainment of disinhibited bursting. This is because disinhibited bursting can be recorded in older spinal cords (Jiang, Carlin et al. 1999) and detection of excitatory effects are more likely, due to lack of inhibition. We found that ventral root evoked entrainment shows a declining trend across early postnatal period (P0-P15) but is detectable even at P24. Pharmacological investigations on younger animals (≤ P8) showed that ventral root evoked entrainment can be enhanced by dopamine D2 receptor antagonism as well as mGluR1 agonism.

## Materials and methods

### Animals

All experiments were carried out in compliance with the National Institute of Neurological Disorders and Stroke Animal Care and Use Committee (Animal Protocol Number 1267-18). Experiments were performed on Swiss Webster WT (Taconic Laboratory) mice from the day of birth to postnatal day 40 (P0–P39).

### Surgical procedures

The mice were anesthetized with isoflurane, followed by decapitation and evisceration. To promote the survival of older spinal cord preparations, we followed the protocol developed to examine motor activity generated in mice of weight-bearing age using the isolated cord preparation (Jiang, Carlin et al. 1999). In brief, the tissue was placed in a dissecting chamber and continuously perfused with ice-cold solution, comprising (in mmol/L): 188 sucrose, 25 NaCl, 1.9 KCl, 10 MgSO_4_, 0.5 NaH_2_PO_4_, 26 NaHCO_3_, 1.2 NaH_2_PO_4_, 25 glucose, bubbled with 95 % O_2_/ 5 % CO_2_. A ventral laminectomy was performed to expose the spinal cord which was transected at the thoracic level (T5-T8). The dorsal and ventral roots were isolated over the lumbar segments and the entire cord was removed from the vertebral column together with the attached roots and transferred to the recording chamber and continuously superfused with artificial cerebrospinal fluid (aCSF; concentrations in mM: 128 NaCl, 4 KCl, 1.5 CaCl_2_, 1 MgSO_4_, 0.5 NaH2PO_4_, 21 NaHCO_3_, 30 d-glucose) bubbled with 95 % O_2_ – 5 % CO_2_. The aCSF was maintained at ambient temperature and the cord allowed to acclimatize for an hour.

### Spinal cord preparations

**Fig. 1:**
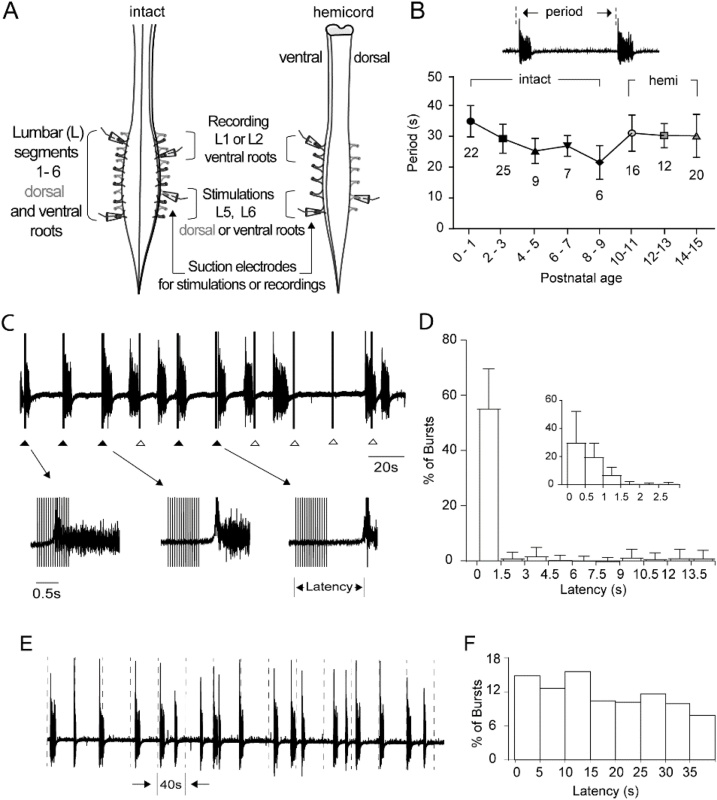
Characteristics of disinhibited bursting and entrainment by ventral root stimulations. A. Schematic of the spinal cord preparations used. **B.** The period of the disinhibited bursting is plotted for P0 – P15. The definition of the period is shown over the plot. Numbers on the plot indicate the number of intact or hemicord preparations used at each age. **C.** An example of a series of stimulus trains applied to the ventral root of a P0 cord to entrain disinhibited bursting. Filled triangles below the trace indicate instances of entrainment. Open triangles indicate stimulus trains that did not produce entrainment. Arrows indicate entrained bursts at a faster timescale (lower panel). **D.** Distribution of latencies from stimulus for a sequence of stimulus trains applied to entrain disinhibited bursting. Data from 22 ventral roots in 15 mice, ages P0 – P3. The inset plot shows the distribution of latencies from 0 to 3 seconds at enlarged timescale. **E.** A short segment of disinhibited bursting record from a P3 cord showing when the assumed stimuli (dashed lines) would have been applied. **F.** Distribution of latency (time from the dotted line to the next burst) for the record in panel E. 5.8 % of the latencies occurred from 0 to 1.5 s for the inter-train interval of 40 s.

We used 2 spinal cord preparations: From birth to 10 days (P0-P9), intact preparations spanning thoracic segments 5–8 (${\text{T}}_{\text{5-8}})$ to cauda equina were used (Fig. 1A). From 11–40 days (P10-P39), hemicord preparations (midsaggital hemisections) spanning thoracic segments 5–8 (${\text{T}}_{\text{5-8}})$ to cauda equina were used (Fig. 1A).

### Data Acquisition and analysis software

Population motoneuron electrical activity was recorded from ventral roots fitted firmly with plastic suction electrodes. The recorded signals were filtered (0.1–3 kHz), digitized at 10 kHz (Digidata 1322A, Molecular Devices) and stored on a computer. Episodes of data were acquired using Clampex (Molecular Devices) and analyzed off-line with Clampfit (Molecular Devices) and Excel (Microsoft).

### Entrainment of the disinhibited bursting

For ventral root evoked entrainment of disinhibited bursts produced by bath-application of bicuculline (20 μM, Sigma) and strychnine (5 μM, Sigma) we used a sequence of stimulus trains applied to a ventral root. The ventral roots were stimulated with repeating trains of 15 pulses at 20 Hz (Stimulus intensity 60 μA, pulse duration 300 μs). The inter-train interval was adjusted to be just shorter than the average period of the disinhibited bursting for each experiment. For disinhibited bursting with an average period between 20−29 s the inter-train interval was 19−24 s; for average period of 30−39 s the inter-train interval was 24−32 s; for average period between 40−49 s the inter-train interval was 34−40 s. Each trial, across experiments and age-groups, comprised of a sequence of 30 successive stimulus trains, with an inter-train interval determined as described above. For dorsal root-evoked entrainment, a single stimulus (duration 300μs; amplitude 5–15 μA) was used with the stimulus intensity defined as that which entrained a burst for each of 10 successive stimuli. Unlike ventral root trials, the inter-train interval for dorsal root stimulations was 10 s.

The latency of onset of the L1/L2 ventral root bursts following ventral root stimulation (L5/6) was characterized, and an upper latency (1.5 s) to define entrainment was established (see results, Fig. 1). Burst onset was defined to be when the neurogram exceeded the mean + 2 standard deviations of the recording before the stimulus. Entrainment was quantified as:

$$\text{E}\text{n}\text{t}\text{r}\text{a}\text{i}\text{n}\text{m}\text{e}\text{n}\text{t}\;(\text{\%})\;=\frac{\text{n}\text{u}\text{m}\text{b}\text{e}\text{r}\;\text{o}\text{f}\;\text{b}\text{u}\text{r}\text{s}\text{t}\text{s}\;\text{e}\text{n}\text{t}\text{r}\text{a}\text{i}\text{n}\text{e}\text{d}}{30\;\text{s}\text{t}\text{i}\text{m}\text{u}\text{l}\text{a}\text{t}\text{i}\text{o}\text{n}\text{s}}\text{x}\;100\;$$

For characterization of ventral root evoked entrainment from P0 – P15, we stimulated all the left and right lumbar ventral roots of segments 5 and 6, in both intact and hemicord preparations. For all other experiments, one or more of the lumbar segments 5 or 6 ventral or dorsal roots were examined.

In experiments that involved 2 trials, we determined the normalized change in entrainment for trial 2 in relation to trial 1 (see Fig. 4, Fig. 5). The normalized change in entrainment was quantified as:

$$\text{N}\text{o}\text{r}\text{m}\text{a}\text{l}\text{i}\text{z}\text{e}\text{d}\;\text{c}\text{h}\text{a}\text{n}\text{g}\text{e}\;\text{i}\text{n}\;\text{e}\text{n}\text{t}\text{r}\text{a}\text{i}\text{n}\text{m}\text{e}\text{n}\text{t}\;(\text{\%})\;=\frac{\text{e}\text{n}\text{t}\text{r}\text{a}\text{i}\text{n}\text{m}\text{e}\text{n}\text{t}\;(\text{\%},\;\text{t}\text{r}\text{i}\text{a}\text{l}\;2)}{\text{e}\text{n}\text{t}\text{r}\text{a}\text{i}\text{n}\text{m}\text{e}\text{n}\text{t}\;(\text{\%},\;\text{t}\text{r}\text{i}\text{a}\text{l}\;1)}\text{x}\;100$$

$$=\frac{\frac{\text{n}\text{u}\text{m}\text{b}\text{e}\text{r}\;\text{o}\text{f}\;\text{b}\text{u}\text{r}\text{s}\text{t}\text{s}\;\text{e}\text{n}\text{t}\text{r}\text{a}\text{i}\text{n}\text{e}\text{d}\;(\text{t}\text{r}\text{i}\text{a}\text{l}\;2)}{30\;\text{s}\text{t}\text{i}\text{m}\text{u}\text{l}\text{a}\text{t}\text{i}\text{o}\text{n}\text{s}}\;\text{x}\;100}{\frac{\text{n}\text{u}\text{m}\text{b}\text{e}\text{r}\;\text{o}\text{f}\;\text{b}\text{u}\text{r}\text{s}\text{t}\text{s}\;\text{e}\text{n}\text{t}\text{r}\text{a}\text{i}\text{n}\text{e}\text{d}\;(\text{t}\text{r}\text{i}\text{a}\text{l}\;1)\;}{30\;\text{s}\text{t}\text{i}\text{m}\text{u}\text{l}\text{a}\text{t}\text{i}\text{o}\text{n}\text{s}}\;\text{x}\;100}\text{x}\;100$$

$$=\frac{\text{n}\text{u}\text{m}\text{b}\text{e}\text{r}\;\text{o}\text{f}\;\text{b}\text{u}\text{r}\text{s}\text{t}\text{s}\;\text{e}\text{n}\text{t}\text{r}\text{a}\text{i}\text{n}\text{e}\text{d}\;(\text{t}\text{r}\text{i}\text{a}\text{l}\;2)}{\text{n}\text{u}\text{m}\text{b}\text{e}\text{r}\;\text{o}\text{f}\;\text{b}\text{u}\text{r}\text{s}\text{t}\text{s}\;\text{e}\text{n}\text{t}\text{r}\text{a}\text{i}\text{n}\text{e}\text{d}\;(\text{t}\text{r}\text{i}\text{a}\text{l}\;1)}\text{x}\;100$$

### Drugs

We investigated if the neurotransmitter and neuromodulatory systems known to effect motoneuron excitatory actions on motor system could alter the efficacy of ventral root evoked entrainment. We investigated the effects of the following drugs on ventral root evoked entrainment: acetylcholine esterase inhibitor donepezil hydrochloride (DH; 1 μM, Tocris), group I mGluR agonist (R,S)-3,5-dihydroxyphenylglycine (DHPG; 1–2.3 μM, Tocris), glutamate transport inhibitor DL-Threo-β-Benzyloxyaspartic acid (DL-TBOA; 10 μM, Tocris), D2 dopamine receptor selective antagonist (L-741626; 4.5–12 μM, Tocris).

Drug experiments consisted of a trial (30 stimuli) of ventral root evoked entrainment of the disinhibited bursts (referred to as trial 1, control), followed by a gap of 15−75 min followed by addition of drugs and a gap of another 15–30 minutes before commencing another trial. The same stimulus parameters were used for trials before and after addition of drugs. If the frequency of the rhythm changed after addition of drugs, the inter-train interval was adjusted accordingly. Drug experiments were carried out from P0 – P8 using intact cord preparations.

Data are expressed as means and standard deviations. All statistical analysis was performed using Prism (GraphPad software, version 8.0.1; La Jolla, CA, USA). Statistical differences between experimental conditions were measured using paired or unpaired parametric or non-parametric tests. Normality of the data were determined using one or more of the following tests depending on the number of samples (n) in the data: Anderson-Darling test, D’Agostino and Pearson test, Shapiro-Wilk test, Kolmogorov-Smirnov test. If the groups were normally distributed paired or unpaired t-tests were employed. If groups were not normally distributed non-parametric tests such as Mann-Whitney, Kolmogorov-Smirnov or Wilcoxon tests were used.

## Results

### Defining ventral root evoked entrainment of disinhibited bursting

To generate disinhibited bursting (example in Fig. 1E) we applied bicuculline (20 μM) and strychnine (5 μM) to the experimental bath (Bonnot, Chub et al. 2009). The rhythm comprised bursts that are synchronized across multiple segments on both sides of the cord. To establish the appropriate inter-train interval for applying the stimulus trains to the ventral roots, the average interval between disinhibited bursts was determined for each experiment. We determined the mean period of disinhibited bursts from P0 to P15 (Fig. 1B). The inter-train interval was adjusted to be just shorter than the average interval between disinhibited bursts for each experiment (see Methods).

We sought to determine the criterion for when a burst was entrained by a stimulus applied to the ventral root, and when a ventral root could be classified as entrainable. To do so, the distribution of latencies of burst onset when applying a sequence of stimulus trains to the ventral root were plotted (Fig. 1C). The distribution of latencies was concentrated between 0 and 1.5 s (Fig. 1D). We compared this distribution to one derived from trials in which no stimulus was applied (Fig. 1E). Plots showing the distribution of latencies from the assumed stimuli are shown in Fig. 1F. We found, as expected a flat distribution for assumed stimuli of 40 s inter-train interval, with only 5.8 % of the bursts within the latency range 0–1.5 s. For these reasons, we classified ventral roots as entrainable if they showed > 7 % entrainment, i.e., > 2 bursts entrained out of 30 stimulus trains, and latency from the stimulus to burst onset is 1.5 s or less.

### Characterization of ventral root evoked entrainment from P0-P15

**Fig. 2:**
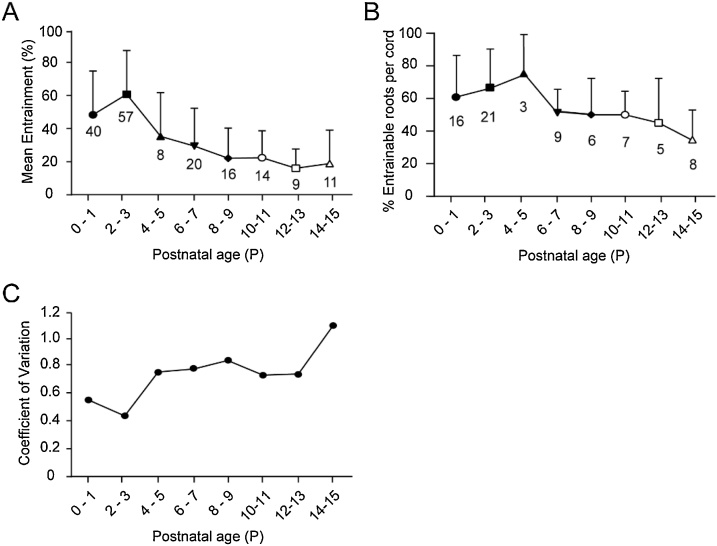
Characterization of ventral root evoked entrainment from P0 to P15. A. Changes in mean value of entrainment at the different ages. Numbers on the plot indicate the number of ventral roots used at each age. **B**. Changes in percentage of entrainable roots per cord at the different ages. Numbers on the plot indicate the number of animals used at each age. **C.** Change in the coefficient of variation for the entrainment data at the different ages. Error bars are SD. P0-P9 are intact cord preparations. P10-15 are hemicord preparations.

In the next set of experiments, we characterized the changes in ventral root evoked entrainment of disinhibited bursts from P0 to P15. Ventral root evoked entrainment is defined as the percentage of bursts that are entrained (latency of burst onset 1.5 s or less) in a sequence of 30 applied stimulus trains. To characterize ventral root evoked entrainment, we used two measures: mean entrainment and percentage of entrainable roots per cord. Mean entrainment is the average entrainment of all entrainable roots (> 7 % entrainment). The percentage of entrainable roots per cord is the percentage of roots out of 4 stimulated per cord (see Methods) that showed entrainment, cords that showed at least one entrainable root were included in the analysis. These results are illustrated in Fig. 2. The mean entrainment reached a maximum at P2-3 when 61.4 ± 27 % of the bursts/trial were entrained (n = 57 roots, 21 animals) and declined to 18.8 ± 20.5 % at P14-15 (n = 11 roots, 8 animals). The decline in mean entrainment across the age-groups was statistically significant (Kruskal-Wallis test, P < 0.0001). The percentage of entrainable roots per cord reached a maximum at P4-5 when 75 ± 25 % of the ventral roots/cord showed entrainment (n = 3 animals) and declined to 34.4 ± 18.6 % at P14-15 (n = 8 animals). It was noticed that entrainment (%) produced by ventral roots even from the same animal could be variable and could range from no entrainment (≤ 7 % entrainment) to high entrainment (upto 100 %). In addition to the changes in entrainment, we examined the coefficient of variation and found that it reaches a minimum of 0.44 at P2-3 and increased to 1.09 at P14-15 (Fig. 2C). Earlier studies have noted the variability of the excitatory effects of motoneurons across spinal cords and even across ventral roots in the same cord (Machacek and Hochman, 2006; Bonnot, Chub et al. 2009; Humphreys and Whelan, 2012) and the present results indicate that this increases with age.

### Oldest ages when disinhibited bursts can be entrained by ventral and dorsal root stimulations

**Fig. 3:**
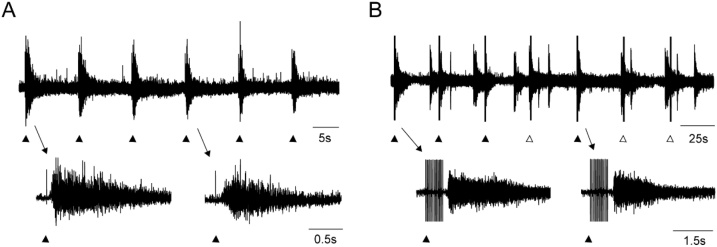
Entrainment of disinhibited bursting at the oldest ages. A. Example of dorsal root evoked entrainment in a P38 hemicord. Arrowheads indicate the sequence of single stimulus (5 μA) applied to the L5 dorsal root. **B.** Example of ventral root evoked entrainment in a P24 hemicord. Arrowheads indicate a sequence of stimulus trains applied to the L5 ventral root. **A and B**. Filled arrowheads below the traces indicate instances of entrainment. Open arrowheads indicate that there was no entrainment. Arrows indicate entrained bursts at a faster timescale (lower panel).

The decline in ventral root evoked entrainment with age could be due to decreased viability of the cords. Tissue oxygenation could potentially get compromised as the cord increases in size and becomes more myelinated with age. Even at the oldest ages we tested (P37-39, n = 3 dorsal roots, 3 animals) we could elicit disinhibited bursting and evoke robust entrainment (100 % entrainment, P38 example in Fig. 3A). Efficacy of dorsal root evoked entrainment at oldest ages remained unchanged from that at age-groups P14-15 and P24 (100 % entrainment, examples shown in Supp. Fig. 1, 3 dorsal roots from 3 animals). Stimulations of ventral roots, in P37- 39 preparations, did not evoke entrainment. Thus, the decline in ability of ventral root stimulations to evoke entrainment with age may not be due to reduced tissue viability.

For ventral root stimulations the oldest age at which we systematically investigated entrainment was in P24 mice (example in Fig. 3B). Entrainment was obtained in 3 of the 12 ventral roots tested with a mean entrainment of 12.3 % (n = 12 ventral roots, 3 animals).

### Decay of entrainment with time

**Fig. 4:**
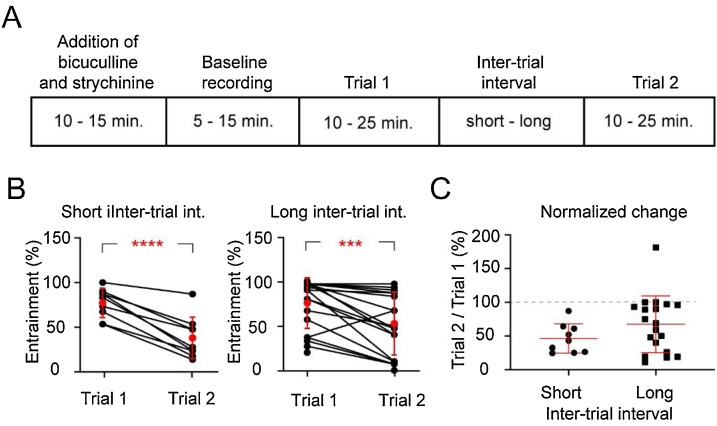
Ventral root evoked entrainment decays following a 30-stimulus trial and partly recovers. A. Experimental protocol to assess time-dependent changes in entrainment. **B.** Plots showing change in entrainment for each experiment. Entrainment values for the two trials for each root are joined by lines, average of entrainment values for each trial are indicated by red filled circles and error bars indicate the standard deviation. Data for short inter-trial interval includes 9 ventral roots from 6 animals aged P0-P5, and that for long inter-trial intervals include 19 ventral roots from 14 animals aged P0-P4. **C.** Plot showing normalized change of entrainment for the experiments with short and long inter-trial intervals.

In previous work it has been noted that, for a given ventral root, entrainment of disinhibited bursting decays with time (Machacek and Hochman, 2006). Our results too reveal that there is a decay in entrainment. In order to quantify the decay and to determine if there is any recovery, we compared experiments with 2 trials each, with short and long inter-trial intervals, where each trial comprised of a sequence of 30 stimulus trains (Fig. 4A). For experiments with short inter-trial intervals the mean interval across experiments was 33.1 ± 3.4 min (9 roots, 6 animals). We found that for shorter inter-trial intervals the mean entrainment for trial 1 was 77 ± 16.6 % and declined to 37.4 ± 23.5 % for trial 2 (Fig. 4B). The reduction in entrainment for trial 2 was statistically significant (*P* <  0.0001, paired *t*-test). For experiments with long inter-trial intervals the mean value across experiments was 97.4 ± 8.2 min (19 roots, 14 animals). We found that for long inter-trial intervals the mean entrainment declined from 77.4 ± 25.9 % for trial 1–54.4 ± 34.1 % for trial 2 (Fig. 4B). The reduction in entrainment for trial 2 was statistically significant (*P* =  0.001, paired *t*-test). The mean value of the normalized change in entrainment ((trial 2 / trial 1) *100) for an inter-trial interval of 30 min. was 46.3 ± 21.6 % while that for 90min. was 67.4 ± 42 % (Fig. 4C). These results confirm a decay in entrainability following a stimulus trial, and further reveals that there is some recovery as well. The mechanism for the decay is unknown, but because we observe some recovery occurs over long inter-trial intervals (mean 97.4 min), some underlying resource necessary for entrainment could be diminished and partly replenished over long periods following a trial. In some experiments to analyze temporal decay more than one root were tested from the same animal. In some cases, we notice that both roots do not necessarily show the same extent of decline (normalized change). For instance, adjacent L5 and L6 ventral roots in one animal showed 90 % and 11 % normalized change (trial2/trial1), while in another animal, adjacent L5 and L6 ventral roots it was 100 % and 89 % respectively.

**Fig. 5:**
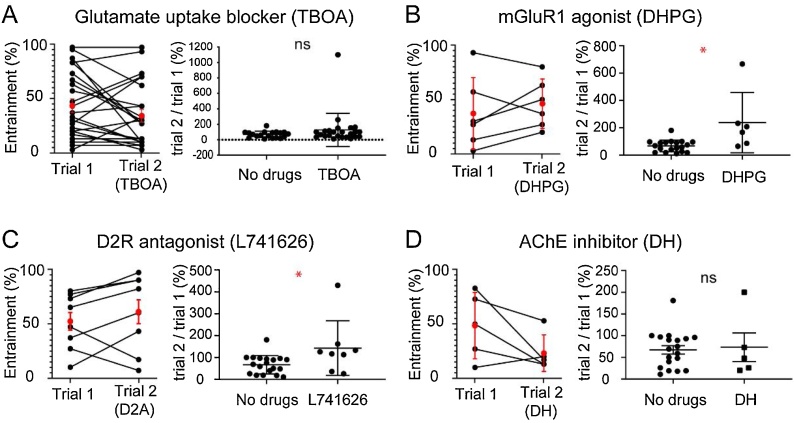
The effects of various drugs on entrainment. A – F. The entrainment values for trials 1 (before drugs) and 2 (after addition of drugs) are shown in the graphs on the left in each panel. The red filled circles indicate the mean and error bars indicate the standard deviation. The normalized change of entrainment (trial 2/ trial 1; mean ± SD) for the experiments with and without drugs (No drugs) are shown in the graphs on the right in each panel. * p < 0.5 **** p < 0.005. See text for further details.

In order to determine the effect of drugs on entrainment it is important to consider that there is decay and recovery of entrainment. For all the drug experiments, we compared the normalized change of entrainment ((trial 2/trial 1) * 100) with the experiments with long inter-trial interval (Fig.4C, normalized change: 67.4 ± 42 %) - referred to as no drugs experiments (Fig. 5, normalized change plot).

### Effect of pharmacology on ventral root evoked entrainment

The excitatory effects of motoneurons have been shown to be glutamatergic (Mentis, Alvarez et al. 2005; Bonnot, Chub et al. 2009; Falgairolle, Puhl et al. 2017), hence we examined glutamatergic agents first. We blocked the operation of excitatory amino acid transporters (EAAT 1–5) using the drug DL-TBOA to establish if increased extracellular glutamate would enhance entrainment (Fig. 5A). In intitial experiments with TBOA at 20−25 u M concentrations it was noticed that there was significant suppression of even dorsal root evoked entrainment that could be reversed upon washout (n = 3 dorsal roots from 3 animals, data not shown), possibly because of persistence of glutamate resulting in saturation of the glutamate receptors. In order to overcome this, a lower concentration of 10 u M was chosen to test the effect on entrainment. The mean entrainment for trial 1 (before drugs) was 43.4 ± 30.7 % and trial 2 (after DL-TBOA 10μM) was 34.2 ± 28.7 % (n = 24 roots, 15 animals). The change in entrainment for trial 2 was not statistically significant (*P* = 0.1305, Wilcoxon test). The mean value of normalized change in entrainment ((trial 2/trial 1) * 100) for DL-TBOA experiments (127.1 ± 214.9 %) was not significantly different (*P* =  0.1919, Mann-Whitney test) from the no drugs experiments (Fig. 5A, normalized change).

Earlier work had shown that mGluR1 antagonists suppress ventral root evoked entrainment in disinhibited cords and abolish ventral root-evoked locomotor like activity (Bonnot et al., 2009). Hence, we examined the effect of metabotropic glutamate receptor 1 (mGluR1) agonist on entrainment. In a previous study (Taccola et al., 2004) the effect of different concentrations of mGluR1 agonist DHPG on frequency of spontaneous disinhibited bursting has been investigated (0.5–1 u M no change in frequency; > 5 u M significant enhancement). Hence, we tested concentrations from 1 to 2.3 u M of DHPG to determine the effect on entrainment. The mean entrainment for trial 1 was 37.2 ± 32.9 % and for trial 2 was 46.2 ± 22.7 % (n = 6 roots, 6 animals; Fig. 5B). The increase in entrainment for trial 2 was not statistically significant (*P* =  0.3477, paired *t*-test). However, the mean value of normalized change in entrainment (trial 2 / trial 1) for DHPG experiments was 237.5 ± 220 % and was significantly greater (*P* =  0.0135, Mann-Whitney test) than that that for no drugs experiments (Fig. 5B, normalized change).

Dopamine has been shown to suppress ventral root evoked entrainment in disinhibited cords and abolish ventral root evoked rhythmic activity, mediating its effect partly through the dopamine D2 receptors (Humphreys and Whelan, 2012). Therefore, we examined if dopamine D2 receptor antagonists (L741626) could enhance entrainment. In a previous study (Sharples, Humphreys et al. 2015) report the effect of D2R antagonist (L-741,626; 6−12 u M) on locomotor rhythm. When applied alone during ongoing locomotor rhythm it has no effect, while applied succeeding the D2R agonist quinpirole, it can nullify the effect of quinpirole. Hence, we investigated the effect of D2R antagonist (L-741,626) on entrainment in concentrations ranging from 4.5 to 12 u M in the present study. We found that the mean entrainment for trial 1 (before drugs) was 52 ± 25.8 % and for trial 2 (after D2R antagonist) it was 60.8 ± 35 % (n = 8 roots, 8 animals; Fig. 5C). The reduction in entrainment for trial 2 was not statistically different from trial 1 (*P* =  0.3011, paired *t*-test). However, the mean value of normalized change in entrainment (trial 2 / trial 1) for the D2 experiments (142.9 ± 125.3 %) was significantly higher (*P* =  0.0225, Mann-Whitney test) than it was for the no drugs experiment (Fig. 5C, normalized change).

We noticed that for the D2 antagonist and the mGluR1 agonist experiments that there was an age-dependent effect on entrainment. For the D2 antagonist, the mean value of the normalized change in entrainment for the youngest animals (P0 and P1) was 31 ± 7.2 % whereas for the older animals (P2 – P5) it was 180.1 ± 123.7 % and the biggest change (430 %) was seen at the oldest age we examined (P5). The age-dependent effects were even more marked for the mGluR1 agonist experiments with the mean normalized change at P1 75.5 ± 14.9 % (2 cords) being much lower than the value (318.6 ± 233.7; 4 cords) in the older animals (P3-P8). Again, the biggest change 666.7 % was seen in the oldest animal studied (P8).

Earlier studies have shown that cholinergic antagonists do not block the excitatory effects of ventral root stimulation on motor networks (Mentis, Alvarez et al. 2005; Bonnot, Chub et al. 2009; Falgairolle, Puhl et al. 2017). Here we examined the effect of an acetylcholine esterase inhibitor (donepezil hydrochloride, 1μM) on entrainment. The drug had no effect on entrainment, consistent with the earlier findings. On washing in the drug, there was an average decrease in mean interval between disinhibited bursts to 51.4 ± 10.2 % of pre-drug value (*P* <  0.05, paired *t*-test), measured in 3 out of 4 experiments. The inter-train interval was adjusted accordingly. The mean entrainment for trial 1 was 48.6 ± 30.6 % (5 ventral roots from 4 animals) and for trial 2 was 23.2± 16.9 % (Fig. 5D). The reduction in entrainment for trial 2 was not statistically different (*P* =  0.125, Wilcoxon test). The mean value of normalized change in entrainment (trial 2 / trial 1) for DH experiments (73.4 ± 73.7 %) was not significantly different (P = 0.868, unpaired *t*-test with Welch’s correction) from that for no drugs data (Fig.5D, normalized change).

We observe that change in entrainment due to effect of drugs can show (trial 2/trial 1): increase, no change, and decrease in entrainment (Fig. 5). This is probably due to the opposing effects of the drugs and temporal decline on entrainment. Hence, the factors most important in interpreting the pharmacology data is the temporal decline (trial2/trial1) of entrainment (no drugs data), and the inter-trial interval for the ‘drugs’ and ‘no drugs’ experiments. The purpose of the Table 1 is to show that for TBOA, mGluR1 agonist, D2R antagonist, DH and no drugs experiments there is variation in inter-trial intervals. For TBOA experiments the mean (± standard deviation) of inter-trial intervals was 71.9 ± 17.7 min. Similarly, for the mGluR1 agonist (DHPG) experiments it was 62.5 ± 19.9 min., D2R antagonist (L741626) experiments it was 48.8 ± 15.5 min., DH (acetylcholine esterase inhibitor) experiments it was 91.2 ± 16 min, and no drugs experiment it was 97.4 ± 8.2 min respectively (see Table 1). The no drugs experiments had the longest mean inter-trial interval. The shorter inter-trail intervals for TBOA, mGluR1 agonist, D2R antagonist in relation to the no drugs experiments likely underestimate the effects of the drugs because less time was available for recovery of entrainment following the first trial. Despite shorter mean inter-trial intervals, we could see significant enhancement in entrainment for D2 antagonists and mGluR1 agonists in relation to the no drugs data. In experiments where more than one root has been investigated per animal, in either the ‘drugs’ or ‘no drugs’ experiments, we did not find a root dependence on the effects observed.

**Table 1:** Inter-trial interval, age-range and number of roots/number of animals.

Experiment	Inter-trial interval (minutes)			Age-range	No. of roots / animals
	minimum	maximum	mean ± st.dev.		
No Drugs	85	114	97.4 ± 8.2	P0 - P4	19 roots / 14 animals
TBOA	45	105	71.9 ± 17.7	P0 - P3	24 roots / 15 animals
DHPG	30	90	62.5 ± 19.9	P1 - P8	6 roots / 6 animals
L741626	30	60	48.8 ± 15.5	P0 - P5	8 roots / 8 animals
DH	74	115	91.2 ± 16	P1 - P4	5 roots / 4 animals

### Discussion

Ventral root evoked entrainment of disinhibited bursts has been examined in several previous studies, but the criterion for classification of bursts as entrained was not defined (Machacek and Hochman, 2006; Bonnot, Chub et al. 2009; Humphreys and Whelan, 2012). The present study formulates the criterion for defining ventral root evoked entrainment (Fig. 1). Based on the criterion formulated, it was found that entrainment declines across early postnatal development. Further, ventral and dorsal root evoked entrainment has been recorded in animals as old as P24 and P39 respectively. Entrainment shows decay after a trial and some recovery over long periods after the trial. We have extended previous pharmacological investigations and shown that both metabotropic glutamate receptor 1 (mGluR1) agonist and dopamine receptor 2 (D2) antagonist enhance ventral root evoked entrainment.

The criterion we have defined for entrainment are that the bursts occur with a latency between 0–1.5 s, and the ventral roots that show >7 % entrainment to be classified as entrainable (Fig.1). Previous work on neonatal mice has shown that the latency to onset of entrained bursts can increase with decrease in interval between stimulus trains (inter-train interval) (Bonnot, Chub et al. 2009). Further the same study has shown that for very short intervals between stimulus trains the latency can increase over 1.5 s. The possible reason for why we do not observe latencies > 1.5 s could be because we have used intervals between successive stimulus trains (inter-train interval) that is just shorter than the average interval between disinhibited bursts for each experiment (see Methods).

### Variability of entrainment and temporal decline

The present study has focused on the L5 and L6 segments and it was found that the coefficient of variation changes from a minimum at P2-P3 to reach a maximum at P14-P15 (Fig. 2). The composition of L5 and 6 ventral roots for a given age-group, say P2-P3, can be considered to remain constant, yet we obtain a range of entrainment values from no entrainment (< 7 %) to high entrainment (upto 100 %). In a previous study (Bonnot, Chub et al. 2009) the rostrocaudal difference in the percentage of roots that can show entrainment; and the variability of the effects of motoneuronal stimulation on entrainment of disinhibited bursting was attributed to the state-dependence of the recurrent excitatory pathway that could be facilitated or unmasked by noradrenaline (Machacek and Hochman, 2006). In the present study entrainment has been enhanced in neonates by dopamine D2 receptor antagonism. A possibility could be that variability of entrainment may partly be due to the excitatory pathway to the rhythm generator, from motoneurons that innervate different ventral roots, being variably affected by the inhibitory influence of dopamine acting via the D2 receptors.

It was reported in a previous study (Machacek and Hochman, 2006) that the ability of ventral roots to entrain decays over time. We have quantified the rate of decay in entrainment after a trial, as well as shown that there is some recovery after a long inter-trial interval. Further, we have shown that temporal decline in entrainment needs to be factored in determining the effect of pharmacology on entrainment. The long duration for recovery of entrainment argues against conventional neurotransmitter depletion and recovery that occur at a much faster rate (Takeuchi, 1958; Liu and Tsien, 1995; Stevens and Tsujimoto, 1995). Further, it has been observed that the rates of temporal decline are variable, even two adjacent ventral roots could show different rates of temporal decline. Implying that the variability of rates of decline of even adjacent ventral roots could be an inherent property of the pathway from motoneurons to rhythm generators. In a previous study investigating the interaction of dorsal and ventral root stimulations on generation of locomotor like activity (Pujala, Blivis et al. 2016), similar results have been found: “inputs from the dorsal and ventral roots do not converge at an early point in their respective pathways to the locomotor CPG. Rather, they appear to converge much closer to the CPG and perhaps within the rhythm generating circuitry itself.”

### Age-related decline in entrainment

We have characterized entrainment and found that it declines from P0-P15, and the variability associated with entrainment increases during the same period. One reason for decline in entrainability with age could be due to decrease in viability as the cord increases in size. We could elicit disinhibited bursting, and dorsal root evoked entrainment in all the P37-P39 hemicord preparations. Entrainment of disinhibited bursting involves eliciting disinhibited bursting (rhythm) and bursts being evoked upon application of sequence of stimulus to dorsal or ventral roots. It has been shown that rhythmogenic networks localized in the ventral regions of the spinal cord produce locomotor like activity (Kjaerulff and Kiehn, 1996) and disinhibited bursting (Bracci, Ballerini et al. 1996). As we can elicit disinhibited bursting and dorsal root evoked entrainment the decline in ventral root evoked entrainment with age may not be due to reduced tissue viability. Previous work has shown that dopamine acting through D2 receptors exert inhibitory influence on motoneuronal effects on rhythm generating networks (Humphreys and Whelan, 2012). Further, we have shown that antagonism of D2 receptors has a facilitatory effect, and the effect increases with age (see Result). Thus, it is possible the decline in entrainment could be due to increased inhibitory influence on motoneuronal actions on rhythm generating networks with age, mediated in part by D2 receptors. The oldest age at which we have systematically investigated and found ventral root evoked entrainment is in P24 mice. The mean entrainment was 12.3 % and in line with the decline we see with age.

A caveat of the study is that while we can elicit ventral root evoked entrainment at older ages, most of the pharmacology experiments were performed in neonates. There are several reasons for this. Entrainment levels are very low to start with in older animals (Fig. 2A), and fewer roots producing entrainment per animal (Fig. 2B). Experiments need to be carried out to investigate the temporal decline of entrainment at the older ages: 1) To quantify the rate of decline of entrainment, 2) How long should the duration between trials be before the roots start to show some recovery of entrainment? Is it the same as in neonates or much longer?, 4) If the inter-trial intervals required are much longer than that in neonates, how would survival/disinhibited bursting be affected in older cords at such long periods of experimentation. Based on the above considerations, careful experiments must be designed to determine if the effects of D2R antagonists or mGluR1 agonists can facilitate entrainment in older animals, preferably in adults.

### Efficiency of Dorsal compared to ventral root evoked entrainment

In our experiments single pulse stimulation of dorsal roots evoke entrainment of disinhibited bursts as has been reported previously (Bracci, Beato et al. 1997; Bonnot, Chub et al. 2009). However, stimulation of ventral roots even by a train of pulses of large intensities are not as effective as dorsal roots in evoking entrainment. In the present study, we have investigated the ability of the L5 and L6 segmental ventral and dorsal roots to evoke entrainment. There are approximately 4 and 5 times as many myelinated fibres, and 13 and 10 times as many unmyelinated fibres, in dorsal compared to ventral roots at the L5 and L6 segments respectively in adult mice (Biscoe, Nickels et al. 1982). The greater numbers of myelinated and unmyelinated fibres could be the reason for robust entrainment of disinhibited bursting by dorsal compared to ventral roots, and stimulus thresholds used in the present study do not likely activate the smallest afferents (Bonnot, Chub et al. 2009).

### Comparison with previous studies

Machacek and Hochman (2006) have reported that “the mean latency of ventral-root-evoked reflexes was −5 ms longer, and more variable in onset, than the monosynaptic reflex evoked with dorsal root stimulation”. The same study also reports that in the same preparations the foci of onset of ventral root evoked reflexes and entrained disinhibited bursts remains the same, in rat spinal cord preparations aged P11-P14. In our study, in preparations aged P11-P14 the mean latency (shortest latency per experiment) to onset of entrained disinhibited bursts was 506 ms (standard deviation 229.6 ms) and is very long in comparison. We do not know the reason for this difference. A possibility could be because we use mice and the quoted study used rats. Another difference between mice and rats that has been previously reported (Bonnot, Chub et al. 2009) and applies to the present study is that the earliest postnatal age at which ventral root evoked entrainment can be elicited in mice is P0; whereas in rats, Machacek and Hochman (2007) report that entrainment cannot be evoked in ages below P9.

### Mode of activation of rhythm generating networks by ventral root stimulation

It has been shown that non-NMDA receptor antagonists block, and mGluR1 receptor antagonists suppress entrainment by ventral roots but not by dorsal roots, and AMPA receptor desensitization blockade enhances entrainment (Bonnot, Chub et al. 2009). However, combined blockade of NMDA and non-NMDA receptors results in abolition of both spontaneous disinhibited and entrained bursting (Bonnot, Chub et al. 2009). Further, dopaminergic action via D2 receptors have been shown to suppress entrainment (Humphreys and Whelan, 2012). We have shown that both D2 receptor antagonism and mGluR1 receptor agonism can enhance entrainment. Taking together, long latencies of entrainment (up to 1.5 s, Fig. 1D) and the ability of D2 and mGluR1 receptors to enhance entrainment, perhaps indicate an important role of metabotropic receptors in addition to the ionotropic receptors in the excitatory pathway from motoneurons to the rhythm generator.

Could the fluctuating rise in extracellular K + triggered directly or indirectly via antidromic firing of motoneurons produce activation of the rhythm generating networks? In a previous study (Marchetti, Beato et al. 2001) it was shown that there is an increase in extracellular K + due to dorsal root stimulations in the rat spinal cord that could evoke alternating rhythmic activity. Further, Bonnot et al. (2009) has shown that ventral root evoked entrainment of disinhibited bursting is abolished by blocker of AMPA/kainate receptors NBXQ, but not dorsal root evoked entrainment (Bonnot, Chub et al. 2009). Furthermore, extending on earlier studies, in the present study it has been shown that there is enhancement of ventral root evoked entrainment of disinhibited bursting in the presence of dopamine D2 receptor antagonists and mGluR1 agonists. Taken together, it seems that fluctuating increase in extracellular K + is not the mechanism for activation of rhythm generating networks by ventral root stimulations.

### Ventral root triggered LOCOMOTOR-LIKE activity (LLA)

We have shown that there is age-dependent and time-dependent decline of entrainment. It remains to be determined if ventral root triggered LLA could be elicited at older ages and if it shows age-, and time-dependent decline in entrainment. Previously, it has been shown that ventral roots have greater efficacy of triggering LLA than entraining disinhibited bursting (Bonnot, Chub et al. 2009). The use of bicuculline at high concentrations has been shown to block calcium-activated potassium channels involved in shaping AHP’s and therefore increase firing rate and excitability (Khawaled, Bruening-Wright et al. 1999). However, the high concentration of bicuculline used may not have a bearing on ventral root evoked entrainment of disinhibited bursting, as ventral roots have lower efficacy of evoking disinhibited bursting compared to triggering LLA.

## Conclusion

The fact that mGluR1 receptor agonism or dopamine D2 receptor antagonism facilitate ventral root evoked entrainment, and the possibility that excitatory interneurons that are contacted monosynaptically by motoneurons mediate the effects of motoneurons on rhythm generating networks (Machacek and Hochman, 2006; Humphreys and Whelan, 2012; Falgairolle, Puhl et al. 2017), could aid further studies to investigate the mechanism producing ventral root evoked entrainment.

## Funding

This research was supported by the Intramural Research Program of the 10.13039/100000065NINDS, 10.13039/501100012264NIH.

## Conflicts of interest

The authors report no declarations of interest.

## CRediT authorship contribution statement

**Chetan Nagaraja:** Conceptualization, Methodology, Formal analysis, Investigation, Writing - original draft, Writing - review & editing.
