# Supplementary material for: Ventral root evoked entrainment of disinhibited bursts across early postnatal development in mice
Source: IBRO Rep. 2020 Oct 27;9:310–8. doi: 10.1016/j.ibror.2020.10.005 (PMC7689330; doi:10.1016/j.ibror.2020.10.005)
Supplement: Supplementary file 1 [file mmc1.docx]

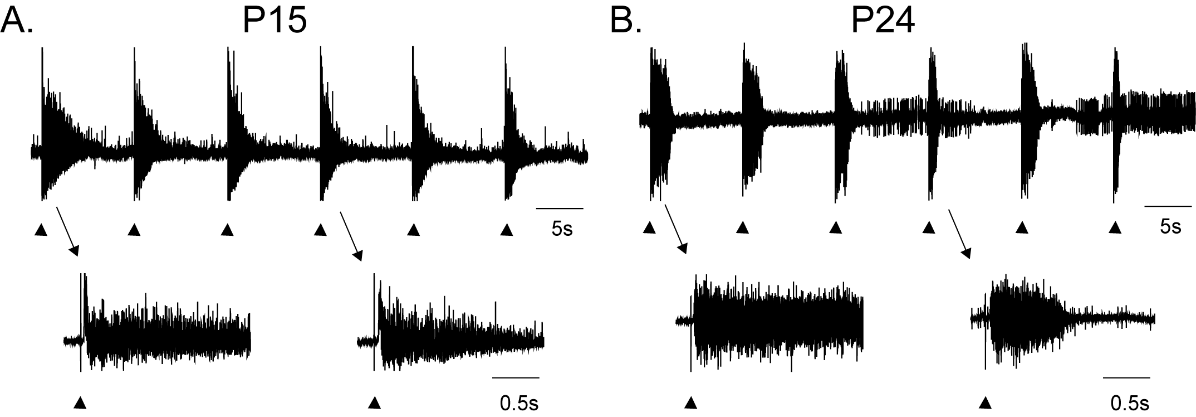


**Supplementary Figure 1. Entrainment of disinhibited bursting evoked by dorsal root stimulations. A.** and **B.** Examples of dorsal root evoked entrainment in P14 (**A**), and P24 (**B**) hemicords. Arrowheads indicate the sequence of single stimulus applied to the L5 dorsal root at stimulus intensity 6.5μA (**A**) and L6 dorsal root at stimulus intensity (11.5μA) (**B**). **A and B**. Filled arrowheads below the traces indicate instances of entrainment. Arrows indicate entrained bursts at a faster timescale (lower
